# Sibling-Derived Cell Lines of Whole Larval Siberian Sturgeon as an In Vitro Model System for Studying Inter-Individual Differences Within the Same Genomic Heritage

**DOI:** 10.3390/cells14242004

**Published:** 2025-12-16

**Authors:** Valeria Di Leonardo, Katrin Tönißen, Julia Brenmoehl, Daniela Ohde, Heike Wanka, Kenneth Benning, Bianka Grunow

**Affiliations:** 1 Fish Growth Physiology, Research Institute for Farm Animal Biology (FBN), 18196 Dummerstorf, Germany; di-leonardo@fbn-dummerstorf.de (V.D.L.); toenissen@fbn-dummerstorf.de (K.T.); 2 Endocrinology of Farm Animals, Research Institute for Farm Animal Biology (FBN), 18196 Dummerstorf, Germany; brenmoehl@fbn-dummerstorf.de (J.B.); ohde@fbn-dummerstorf.de (D.O.); 3 Institute of Physiology, University Medicine, 17475 Greifswald, Germany; heike.wanka@med.uni-greifswald.de; 4 London Fine Foods Group/CellRoe, London SW11 3HB, UK; kennethbenning@londonfinefoods.co.uk

**Keywords:** sibling variation, extracellular acidification rate (ECAR), mitochondrial activity, larval cell line, fish in vitro model

## Abstract

**Highlights:**

**What are the main findings?**

- Five sibling-derived cell lines were successfully generated and maintained for long-term culture, cryopreservation, and transfer.
- The lines exhibited biological variation in cell morphology, mitochondrial activity, and extracellular acidification rates while the gene expression profiles closely resembled those of whole larvae with in vitro adaptations observed for *gapdh*, *vim*, *col1a1*, and *pcna*.

**What are the implications of the main findings?**

- First establishment of larval cell lines from the critically endangered species Siberian sturgeon (*Acipenser baerii*).
- These new larval cell lines provide a powerful in vitro model to explore developmental biology and stress responses in endangered sturgeons.

**Abstract:**

Sturgeons, once resilient enough to outlive dinosaurs, are now critically endangered. All 26 species of Acipenseriformes face extinction due to anthropogenic causes. Despite their ecological and economic significance, sturgeon research lacks essential tools such as larval cell lines; the Cellosaurus database lists only one larval cell line (AOXlar7y from Atlantic sturgeon). Larval stages are key to understand fish development, representing a transitional phase between embryonic and adult life that is highly sensitive to temperature shifts, oxygen depletion and pollution. Larval cell lines therefore provide potential in vitro models for studying development and stress responses in endangered species. This study focused on establishing and initially characterizing five novel larval cell lines from siblings of the Siberian sturgeon (*Acipenser baerii*). The lines proved viable for long-term culture, bio-banking and transfer, displaying different morphologies ranging from epithelial-like to fibroblast-like. Functional assays showed variable mitochondrial activity and extracellular acidification rates. A preliminary targeted gene expression analysis revealed similarity to whole larvae within early passages and in vitro adaptations for certain genes (*gapdh*, *vim*, *col1a1*, *pcna*). These sibling-derived cell lines hold potential as in vitro tools to deeper explore the biology of Siberian sturgeon larvae and support conservation-focused research.

## 1. Introduction

Fish cell lines are of fundamental importance for studying fish biology and exploring biotechnological solutions to improve animal welfare, enhance aquaculture sustainability, and develop species conservation strategies, all in line with the principles of the 3Rs (Replace, Reduce, Refine) for animal experiments. The development of fish cell cultures has significantly contributed to research, environmental toxicology and aquaculture studies [1]. Despite their potential, fish-derived cell lines remain generally underexplored due to the diversity of species and their physiology, with over 36,000 species [2]. Consequently, the lack of standardized, validated protocols for cell line establishment, cultivation, and characterization has limited their use in the past [3].

Sturgeons are heavily exploited for caviar and meat [4]. Yet they represent the most endangered animal group worldwide, according to the International Union for Conservation of Nature (IUCN). All 26 species of the Acipenseriformes have continued to decline since 2010 and are now classified as critically endangered or threatened with extinction [5]. These ancient fish urgently require science-based conservation strategies and cell-level approaches to deepen our understanding and support recovery efforts.

To date, the sturgeon cell lines available in the Cellosaurus database account for only one larval cell line (AOXlar7y from Atlantic sturgeon, *Acipenser oxyrinchus*) [6,7]. This cell line has proven helpful for toxicological and climate change studies [8,9]. The larval stage is a critical and vulnerable phase in fish development, and is marked by high sensitivity to environmental stressors. Understanding these aspects using cell lines is crucial for predicting the health and abundance of wild and farmed fish populations, including fish larvae [10].

Individuals often show sustainable variation in growth and survival even under identical conditions, a pattern observed across species and even among siblings [11]. The variations are linked to multifactorial physiological traits and can persist despite standardized food intake [12]. Some of these variations can be examined at the cellular level [13].

Therefore, this study aims to address the gap in the availability of sturgeon whole-larval cell lines by establishing and characterizing five novel larval cell lines derived from siblings of the species Siberian sturgeon (*Acipenser baerii*).

The development and characterization of these cell lines provide a valuable tool for studying the molecular mechanisms underlying sturgeon larval development, and offers a crucial comparison to identify potential inter-individual differences among siblings. This will enhance our understanding of the biology of this highly endangered species and support the development of innovative, welfare-oriented, and sustainable conservation and aquaculture strategies for sturgeons under climate change and habitat degradation.

## 2. Materials and Methods

### 2.1. Animals

*A. baerii* larvae from the same broodstock, at the yolk sac stage were used in this study and were obtained from Royal Belgian Caviar (Turnhout, Belgium). Larvae were 4 days post-hatching (dph), at a stage where feeding had not yet commenced. The larvae were in good health, exhibiting active movement and no apparent bacterial or fungal contamination.

Larvae used for cell isolation were kept in water on ice, a method known as temperature-induced hypothermia, to reduce nervous activity and metabolism to lessen the stress. Cold, less mobile larvae were restrained and killed immediately by decapitation.

### 2.2. Cell Isolation

Tissue of each larvae was finely minced using scissors during a one to two-minute digestion in a 0.1% trypsin/EDTA solution (Gibco, Paisley, UK) [7]. The digestion was terminated by adding twice the volume of culture medium (Leibovitz-15 Medium (L-15), Gibco, Paisley, UK), supplemented with 20% fetal bovine serum (FBS, PAN-Biotech) and 1% (*v*/*v*) penicillin/streptomycin (P/S, Gibco). After centrifugation (130× *g*; 5 min), the cells were resuspended in the culture medium with additional antibiotics (Gentamycin: 0.1 mg/mL, Gibco, Paisley, UK; Kanamycin: 0.5 mg/mL, Gibco, Grand Island, NY, USA), along with an antimycotic agent (Amphotericin: 250 μg/mL, Gibco, Paisley, UK). Cells were seeded into one well of a 6-well plate (TPP, Techno Plastic Products AG, Trasadingen, Switzerland) and incubated at 22 °C. The primary culture remained undisturbed for 48 h, and then washed with 1× Dulbecco’s Buffered Saline (PBS, PAN Biotech, Aidenbach, Germany) and the medium was replaced. During the first two weeks, the medium was replaced every two days, and the antibiotics mentioned above were added to the culture. After that, a medium exchange occurred every third or fourth day without the additional antibiotics, except for 1% P/S. Once the cells reached confluence, they were sub-cultured at a 1:2 ratio by standardized protocols [8]. For the first 10 passages (P), cells were cultured in L-15 medium with 20% FBS, then in 15% FBS for two passages, and from P12 onward in 10% FBS to reduce serum use and standardize the cultivation.

### 2.3. Cell Line Analysis

Cell isolations were performed from 12 larvae, resulting in the establishment of 7 cell lines. Due to low proliferation rates, two of these cell lines originated from three larvae (RBC-ABAlar1, RBC-ABAlar4). The other five cell lines originated from individual animals (RBC-ABAlar7, RBC-ABAlar8, RBC-ABAlar9, RBC-ABAlar11, and RBC-ABAlar12), which were used in this paper for more detailed analysis. Cell morphology was monitored by regular phase-contrast imaging using an inverted phase-contrast microscope (Motic^®^ AE2000, Wetzlar, Germany), a Moticam 5 Plus camera and Motic Images Plus 3.0 Software (Motic^©^, Wetzlar, Germany). Further adjustments to brightness and contrast were made using Adobe Photoshop CC 2019 (Adobe Inc., San Jose, CA, USA). In parallel, cell number, vitality, and cell size were determined by trypan blue staining in combination with the EVE™ Plus automatic cell counter (NanoEnTek, Gyeonggi-do, Republic of Korea), following manufacturer’s instructions.

To evaluate cryopreservation, two freezing media were tested (FBS: DMSO 9:1; or 10% DMSO, 40% FBS, and 50% culture medium). Trypsinised cells were resuspended in ice-cold freezing medium, transferred to cryo-tubes and cooled to −80 °C overnight in a pre-cooled isopropanol surfaced freezer box. Subsequently, the vials were stored in the liquid nitrogen atmosphere for about a month. For reseeding, the cells were quickly thawed in cell culture medium pre-warmed to room temperature, centrifuged at 130× *g* for 5 min, resuspended, and seeded. Vitality and cell size were measured before and after this process.

### 2.4. Analysis of Metabolic Function

The metabolic function of the RBC-ABAlar cell lines (7, 8, 9, and 12 at P17-22) was determined with the Seahorse XF96 Cell Analyzer (Agilent, Waldbronn, Germany) by measuring the oxygen consumption rate (OCR) using the Mito Stress Test (#103015-100; Agilent). The RBC-ABAlar11 was excluded from the final analysis due to its low proliferation rate and poor attachment to the Seahorse XFe96 cell culture microplate (Agilent).

To evaluate mitochondrial function and cellular metabolic activity, different seeding densities (2500, 5000, and 10,000 cells per well) of RBC-ABAlar cell lines 7, 8, 9, and 12 at different concentrations (1 µM and 2 µM) of the metabolic inhibitors oligomycin and carbonyl cyanide p-trifluoromethoxyphenylhydrazone (FCCP) were tested, with the most reliable results achieved with 5000 cells, and 2 µM oligomycin as well as 2 µM FCCP, which were selected for the final assays.

For metabolic analysis, cells were seeded into eight wells per cell line in a Seahorse XFe96 cell culture microplate, and cultured for 24 h to allow attachment. After replacing the cell culture medium, the cells were cultured for four more days until confluence was reached, and then analyzed. OCR and extracellular acidification rate (ECAR) were measured, and all parameters were calculated as described in detail previously [9].

### 2.5. Mitochondrial Membrane Potential (MMP) Analysis

RBC-ABAlar cells were detached from culture flasks using the standard trypsinization method and washed in 1x PBS. The cell concentration was determined using a hemocytometer, and the cells were resuspended to 5 × 10^5^ cells/mL in fresh medium. Control and treated cells were incubated with 2 µM JC-1 dye (T4069, Sigma-Aldrich now part of Merck KGaA, Darmstadt, Germany) and 2 µM JC-1 dye + 50 µM carbonylcyanide-3-chlorophenylhydrazone (CCCP; C2759, Sigma-Aldrich), respectively, at room temperature for 30 min, protected from light. After incubation, the cells were washed and resuspended in fresh medium. The samples were analyzed in a flow cytometer (Gallios, Beckman Coulter, Krefeld, Germany). A gating strategy was applied to isolate viable cells from debris and aggregates. Cells were then analyzed based on JC-1 fluorescence in the red (590 nm) and green (530 nm) channels.

### 2.6. Analysis of Gene Expression

#### 2.6.1. Selection of Genes and Primer Design

For the gene expression analysis, eight genes were selected (Table 1) with a focus on the regulation of glycolysis with Glyceraldehyde-3-phosphate dehydrogenase (*gapdh*), proliferation with Proliferating cell nuclear antigen (*pcna*) and pluripotency with POU domain, class 5, transcription factor 1 (*pou5f1*), as well as cytoskeleton stability with Spectrin alpha chain non-erythrocytic 1 (*sptan1*), Vimentin (*vim*), Collagen Type I Alpha 1 Chain (*col1a1*) and stress with Heat Shock Transcription Factor 1 (*hsf1*) and Heat shock protein 70 (*hsp70*). Two reference genes, Eukaryotic Translation Elongation Factor 1, Alpha 1 (*eef1a1*) and Ribosomal Protein L6 (*rpl6*) were chosen to normalize the expression data [9,14], and corresponding primers were either designed as described below or obtained from the literature [9].

Sequences for the primer design were obtained from the NCBI GenBank database. If available, gene sequences from sturgeon were used. Otherwise, sequences from *Polyodon spathula* were used. The oligonucleotide primer sequences (Table 1) were designed using the PSQ-Assay Design software (Version 1.0.6, Biotage) and synthesized by Sigma-Aldrich (Merck). The efficiency and specificity of the designed primers were assessed by polymerase chain reaction (PCR) followed by gel electrophoresis and quantitative real-time (qPCR) analyses.

#### 2.6.2. RNA Extraction and Fluidigm PCR

As a positive control, three pools of three larvae were mechanically homogenized (Precellys Evolution, Bertin technologies, Montigny-le-Bretonneux, France) and RNA was isolated using the Trizol-Chloroform precipitation method. The total extracted RNA was purified using the RNeasy^®^ Plus Micro Kit (Qiagen, Hilden, Germany) according to the manufacturer’s instructions.

RBC-ABAlar cells were used for the comparative analysis. Cell pellets were collected from 75 cm^2^ culture flasks at full confluence (on average 1.5 × 10^6^ cells) and stored at −80° C. For each cell line, samples were collected twice: once at P13 and once at a higher passage (P23–34). RNA was isolated with RNeasy^®^ Plus Micro Kit (Qiagen) according to the manufacturer’s instructions and quantified with a NanoDrop One spectrophotometer (Thermo Fisher Scientific, Darmstadt, Germany) using absorbance at 260 nm. RNA purity was assessed by the 260/280 nm absorbance ratio.

The cDNA synthesis was performed with the iScript™ cDNA Synthesis Kit (Bio-Rad Laboratories, Inc., Hercules, CA, USA) following the manufacturer’s protocol.

For a high-throughput analysis of gene expression in the different sample groups, a transcriptional analysis was conducted with quantitative real-time PCR using the Biomark HD system (Standard BioTools, South San Francisco, CA, USA) with GoTaq^®^ qPCR mastermix (Promega Corporation, Madison, WI, USA) containing BRYT^®^ green dye, following the Fluidigm 48.48 Dynamic Array IFC (Standard BioTools) [15]. Each gene and sample were run in duplicate, and Ct-values were obtained using the real-time PCR analysis software (Fluidigm, Version 3.0.2, Standard BioTools). Gene expressions were normalized to the reference genes using DAG Expression software (version 1.0.5.6) [16].

### 2.7. Statistical Analysis

Statistical analyses were performed using GraphPad Prism software, version 10.5.0 (GraphPad Software, Inc now part of Dotmatics, www.graphpad.com). Statistical tests are specified in the figure captions. The choice of statistical test was guided by the data distribution and the underlying test assumptions. Technical replicates were treated as independent values. The statistical difference was intended as * *p* < 0.05, ** *p* ≤ 0.01, *** *p* ≤ 0.001, **** *p* ≤ 0.0001. Data are presented as mean ± SD.

## 3. Results

### 3.1. Cell Culture Development

Cell outgrowth was observed 48h after isolation, forming a confluent monolayer around explants within one week. In the first passages (P0–5), no differences were visible in the isolated cells of the different larvae.

**Figure 1:**
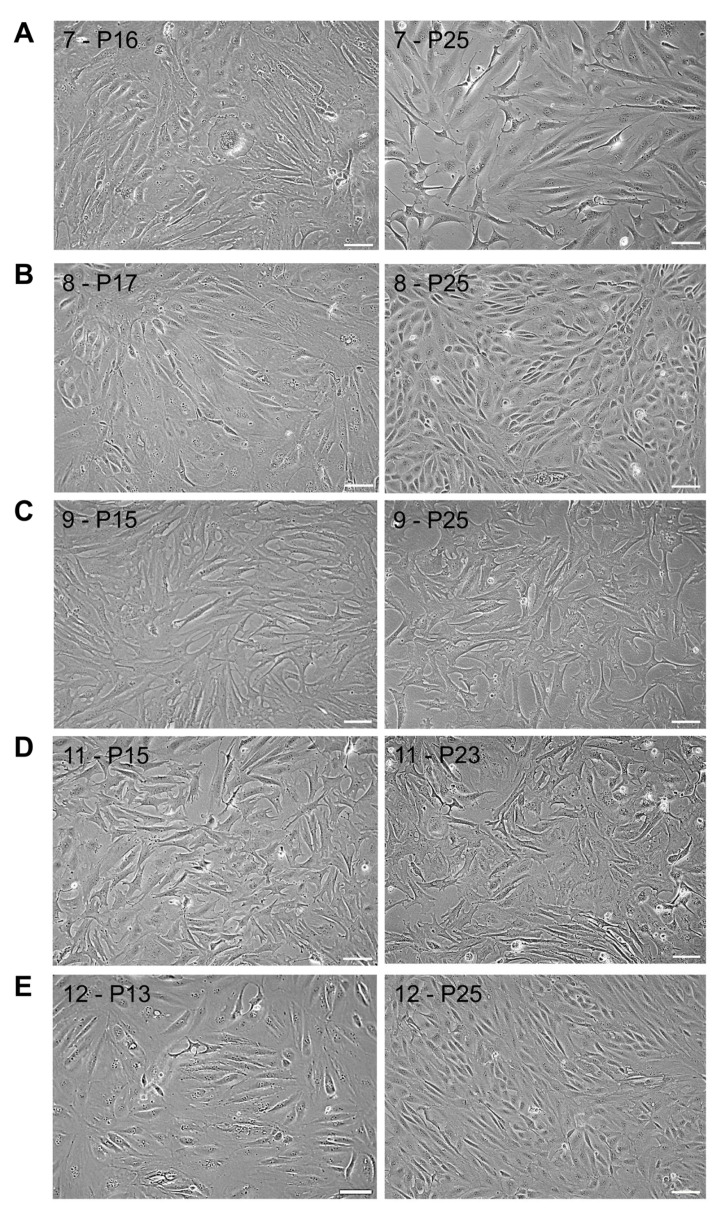
Microscopic overview by phase contrast of RBC-ABAlar cell lines at P13/17 and P23/25. (**A**) RBC-ABAlar7, (**B**) RBC-ABAlar8, (**C**) RBC-ABAlar9, (**D**) RBC-ABAlar11, (**E**) RBC-ABAlar12. Scale bars: 100 µm.

From P2 onwards, cells proliferated as monolayers, and from P5 onwards, cells appeared more homogeneous, with RBC-ABAlar7 showing mixed fibroblast- and epithelial-like morphology (Figure 1A), RBC-ABAlar8 and RBC-ABAlar12 mainly epithelial-like morphology (Figure 1B,E), as well as RBC-ABAlar9 and RBC-ABAlar11 fibroblast-like shape (Figure 1C,D).

**Figure 2:**
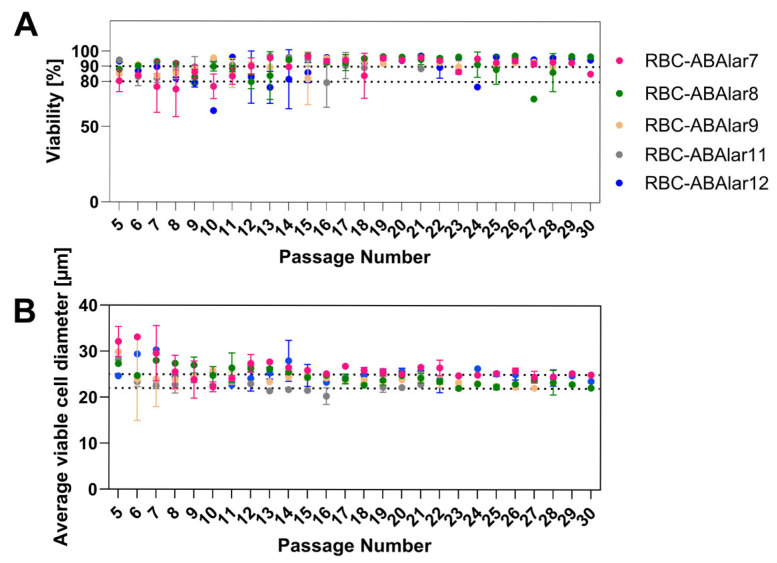
Characteristics of RBC-ABAlar cells at different passage numbers. (**A**) viability with reference lines at 80% and 90%, and (**B**) cell diameter with reference lines at 22 µm and 25 µm, shown for established RBC-ABAlar cell lines 7, 8, 9, 11, and 12. Passage numbers range from 5 to 30. Data are presented as mean ± SD (replicates per passage and cell line: n = 2–5).

Cells were passaged up to 30 times over six months, maintaining high viability (88–90%; Figure 2A) and stable cell size (22–25 µm; Figure 2B) after an initial period of variation.

Cryopreservation from P6 onwards did not affect viability (83–96% in all passages) or morphology when using the two different freezing media.

### 3.2. Metabolic Function of the Cells

**Figure 3:**
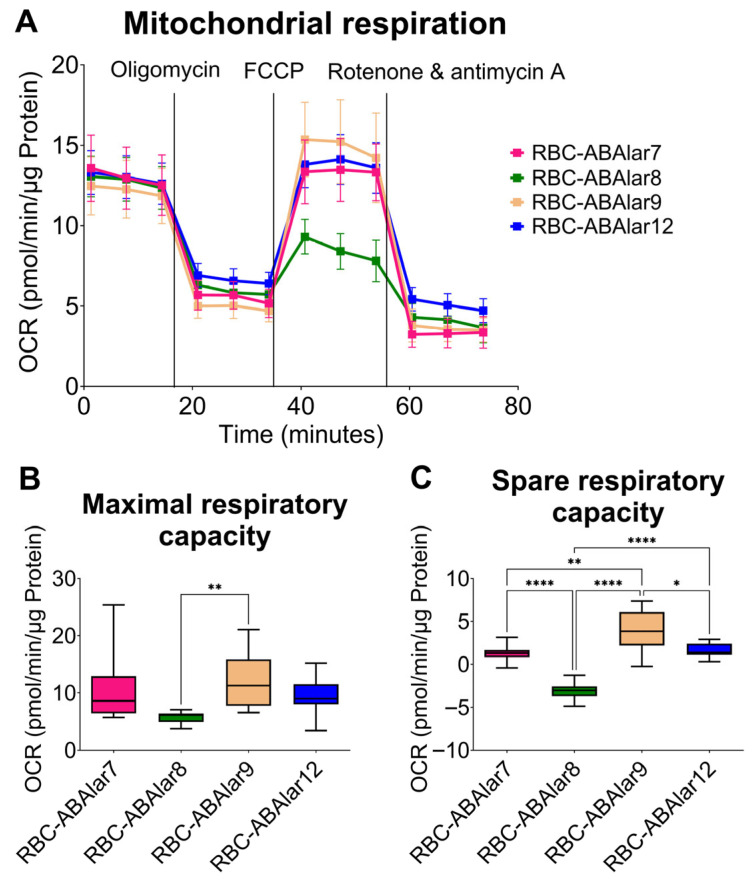
Overview of the Mito Stress Test in RBC-ABAlar cell lines. (**A**) Overview of oxygen consumption rate (OCR) under basal conditions and after administering oligomycin, FCCP, and rotenone/antimycin A in RBC-ABAlar cell lines 7, 8, 9, and 12. Data are presented as mean ± SD (n = 8). (**B**) Maximal respiration and (**C**) spare respiratory capacity of the same cell lines. Data are presented as mean ± SD (n = 8). The Kruskal–Wallis test with Dunn’s correction for multiple comparisons (**B**) and Ordinary One-Way ANOVA with Tukey correction (**C**) were applied for statistical testing. Statistical differences: * *p* < 0.05, ** *p* ≤ 0.01, **** *p* ≤ 0.0001.

To assess cellular metabolism, mitochondrial respiration (indicated by OCR) and glycolysis (indicated by ECAR) were measured. Basal respiration, ATP-linked respiration, proton leak, and non-mitochondrial OCR were comparable across all RBC-ABAlar lines with no significant differences observed (Figure 3A). RBC-ABAlar8 exhibited significantly lower maximal respiratory capacity than RBC-ABAlar9 (Figure 3B) and spare respiratory capacity compared to the other cell lines (Figure 3C). At the same time, RBC-ABAlar9 showed significantly greater spare respiratory capacity than lines 7, 8, and 12.

**Figure 4:**
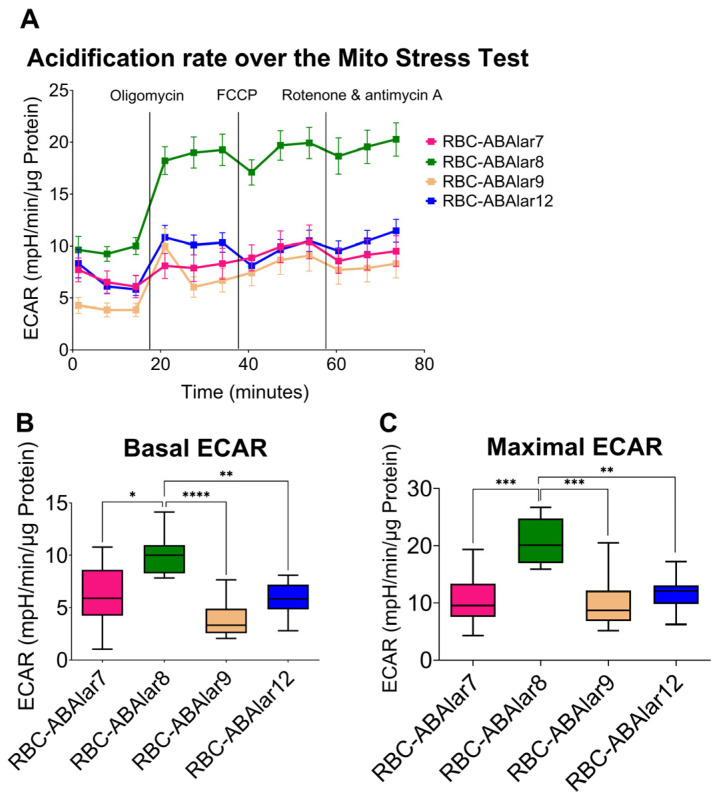
Overview of the extracellular acidification rate (ECAR) in RBC-ABAlar cell lines during the Mito Stress Test. (**A**) Overview of ECAR was measured under basal conditions and following sequential injections of oligomycin, FCCP, and rotenone/antimycin in RBC-ABAlar cell lines 7, 8, 9, and 12. (**B**) ECAR parameter under basal conditions. (**C**) ECAR parameter after oligomycin and FCCP injection. Data are presented as mean ± SD (n = 8). An ordinary one-way ANOVA with Tukey correction for multiple comparisons was used for statistical testing. Statistical differences: * *p* < 0.05, ** *p* ≤ 0.01, *** *p* ≤ 0.001, **** *p* ≤ 0.0001.

The inhibition of mitochondrial ATP synthase by oligomycin led to a significantly stronger glycolytic response in the ABAlar8, as indicated by higher ECAR values, whereas RBC-ABAlar7 showed a lower increase (Figure 4A). Under basal conditions, glycolytic metabolism in RBC-ABAlar8 was higher than in the other cell lines, especially compared to RBC-ABAlar9 (Figure 4B). Also, the oligomycin- and FCCP-induced ECAR increase in RBC-ABAlar8 was even more pronounced (Figure 4C).

**Figure 5:**
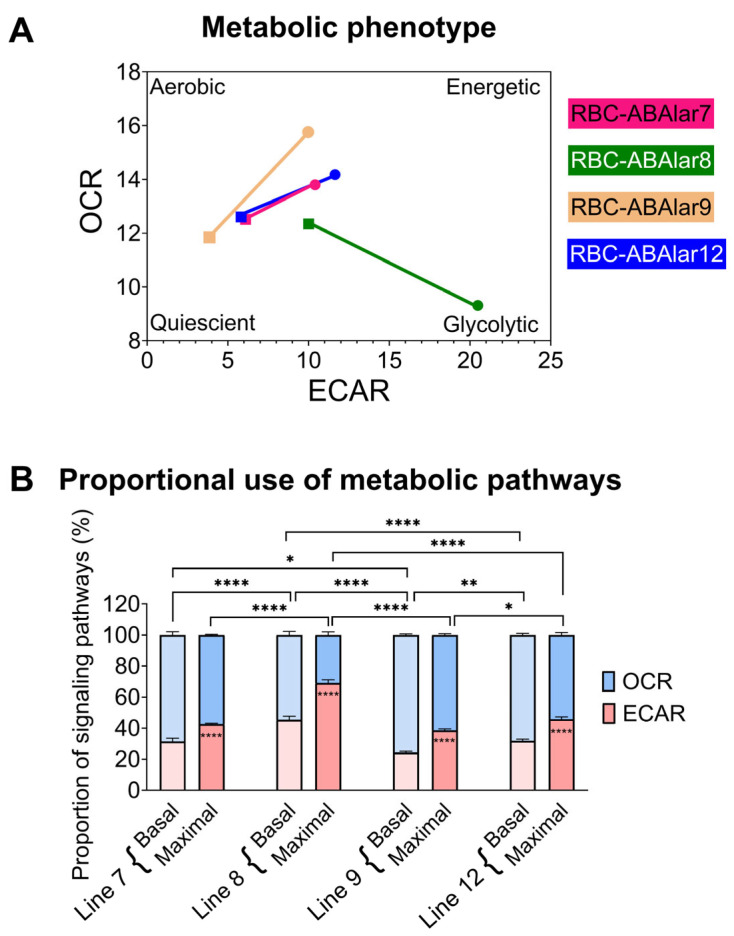
Bioenergetic phenotype of RBC-ABAlar cells. (**A**) The transition of the energy phenotype from basal (square symbol) to FCCP injection-induced maximal conditions (round symbol) was determined by measuring extracellular acidification rate (ECAR) and oxygen consumption rate (OCR) using the Mito Stress Test in RBC-ABAlar cell lines 7, 8, 9, and 12. (**B**) Proportional distribution (%) of OCR and ECAR under basal and maximal conditions. A two-way repeated-measures mixed-effects ANOVA with Šidák correction for multiple comparisons was applied as statistical testing. Data are presented as mean ± SD (n = 8). Internal asterisks indicate the statistical differences between the basal and maximal conditions of each cell line. Statistical differences: * *p* < 0.05, ** *p* ≤ 0.01, **** *p* ≤ 0.0001.

The consequent analysis of the metabolic phenotype demonstrated that metabolism depended on both glycolytic and oxidative metabolic pathways across all cell lines (Figure 5A). Metabolic profiling confirmed that RBC-ABAlar7, 9, and 12 were predominantly oxidative, while RBC-ABAlar8 displayed a glycolysis-dominated phenotype, particularly under uncoupling oxidative phosphorylation by FCCP. The proportional distribution of glycolytic to oxidative catabolic pathways confirmed the dominance of oxidative pathways in RBC-ABAlar cells 7, 9, and 12 under both basal and stressed conditions (Figure 5B). In contrast, RBC-ABAlar8 relied on oxidative and glycolytic pathways at baseline and shifted towards glycolysis under stress, whereas RBC-ABAlar9 showed the most oxidative phenotype (Figure 5A,B).

### 3.3. Flow Cytometry and MMP

Since after uncoupling electron transport in the respiratory chain and oxidative phosphorylation, the FCCP-induced OCR did not exceed the basal OCR, especially in the RBC-ABAlar8 line, flow cytometry analysis was performed to confirm the impact of FCCP on the MMP of the studied cell lines.

**Figure 6:**
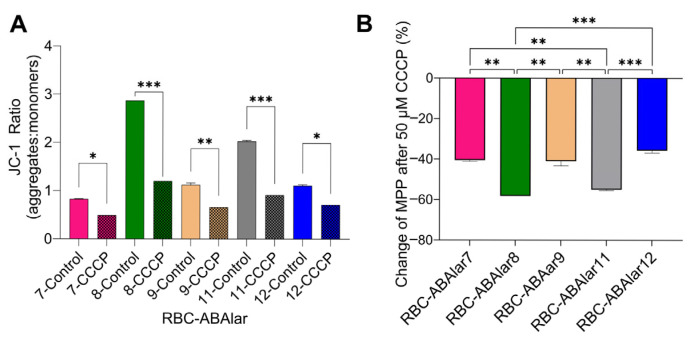
(**A**) JC-1 ratio (aggregates/monomers or red/green fluorescence) measured in untreated (control) and CCCP-treated cells. (**B**) Percentage reduction (%) of cells’ MMP after CCCP administration. Data are presented as mean ± SD from 5 × 10^5^ cells per RBC-ABAlar cell lines 7, 8, 9, 11, and 12. A two-way ANOVA (**A**) and a one-way ANOVA with Tukey test for multiple comparisons (**B**) were applied. Statistical differences: * *p* < 0.05, ** *p* ≤ 0.01, *** *p* ≤ 0.001.

The CCCP treatment, comparable in its effect to FCCP, significantly reduced the JC-1 ratio in all cell lines, resulting in effective mitochondrial membrane depolarization (Figure 6). The JC-1 ratio was halved or reduced even further (Figure 6A). The percentage reduction (%) of MMP values varied significantly between the cell lines (Figure 6B). RBC-ABAlar8 had the most pronounced response to CCCP, followed by RBC-ABAlar11. In contrast, RBC-ABAlar7, 9, and 12 exhibited less marked reductions.

### 3.4. Gene Expression Analysis

**Figure 7:**
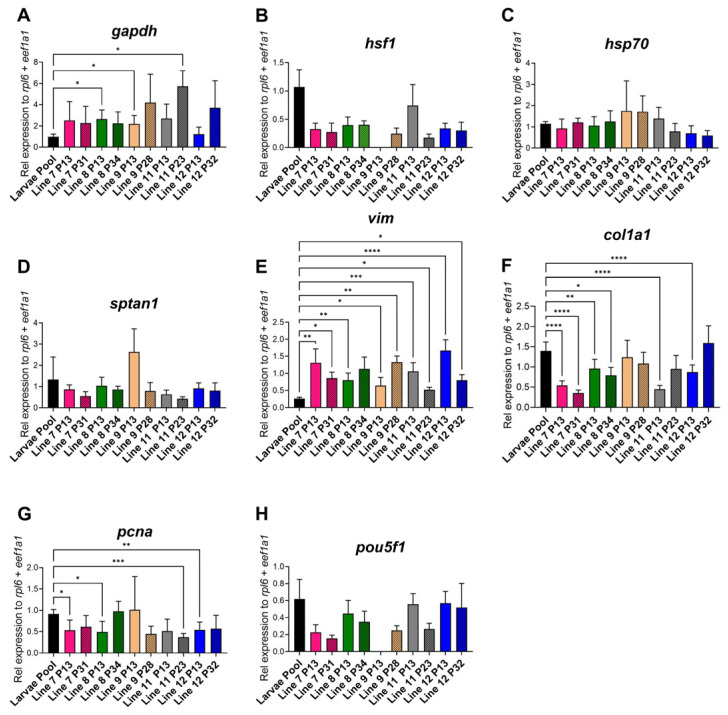
Relative gene expression quantification and comparison between whole larvae and cell lines. (**A**) Glyceraldehyde-3-phosphate dehydrogenase (*gapdh*), (**B**) Heat Shock Transcription Factor 1 (*hsf1*), (**C**) Heat shock protein 70 (*hsp70*), (**D**) Spectrin alpha chain non-erythrocytic 1 (*sptan1*), (**E**) Vimentin (*vim*), (**F**) Collagen Type I Alpha 1 Chain (*col1a1*), (**G**) Proliferating cell nuclear antigen (*pcna*), (**H**) POU domain class 5 transcription factor 1 (*pou5f1*). Larvae pool: black. RBC-ABAlar cell lines: 7 (pink), 8 (green), 9 (yellow), 11 (gray), and 12 (blue). P13 (homogenous color) and later passages (diagonal line pattern). Larvae pool biological replicates: n = 3 × 3 larvae and technical replicates n = 6. RBC-ABAlar cell lines in P13: technical replicates n = 8. RBC-ABAlar cell lines in later passages: technical replicates n = 4. Data are expressed as mean ± SD. Welch and Brown-Forsythe versions of one-way ANOVA with the Dunnett T3 test for multiple comparisons were applied. Statistical differences: * *p* < 0.05, ** *p* ≤ 0.01, *** *p* ≤ 0.001, **** *p* ≤ 0.0001.

To confirm similarities between the RBC-ABAlar cell lines (7, 8, 9, 11, and 12) and the larval organism, gene expression profiles of cells and a larval pool from siblings of the same generation were compared. The glycolysis-related gene *gapdh* was significantly higher in most cell lines compared to the larval pool, particularly in RBC-ABAlar 8 (P13), 9 (P13), and 11 (P23) (Figure 7A). The heat shock markers *hsf1* and *hsp70* were homogeneous with no significant differences, except for the outlier RBC-ABAlar9 at P13, which was excluded from the analysis (Figure 7B,C). The structural gene *sptan1* exhibited no significant differences between larvae and the cell lines, with a minor decrease in later passages, while the cytoskeleton-associated gene *vim* was significantly higher expressed in all the cell lines (Figure 7D,E). In later passages, the expression continued to increase in RBC-ABAlar8 and 9, while it decreased in the other lines (Figure 7E). The expression of *col1a1* was significantly reduced in RBC-ABAlar 7 and 8 (both passages) and in RBC-ABAlar 11 and 12 (P13), with later passages showing variable trends (Figure 7F). The proliferation marker *pcna* was significantly lower expressed in RBC-ABAlar 7, 8, 12 at P13, and in RBC-ABAlar11 at P23, but stable or increased in RBC-ABAlar8 cells at P32 and RBC-ABAlar9 (P13), (Figure 7G). The expression of the pluripotency marker *pou5f1* showed no significant differences compared to the larvae pool (Figure 7H). Like for the *hsf1* gene, RBC-ABAlar9 (P13) was excluded from the analysis due to technical abnormalities.

## 4. Discussion

In this study, five new cell lines from siblings of the endangered species Siberian sturgeon (*A. baerii*) were established and characterized. These new cell lines expand larval cell models, enable species- and individual-specific studies, and support the 3Rs principles for reducing animal testing. They also offer potential for better environmental protection and sustainable aquaculture, as the Siberian sturgeon is globally farmed for meat and caviar production [17,18,19,20].

### 4.1. Cell Culture Development and Gene Expression

RBC-ABAlar cell lines showed stable growth and cell sizes in later passages, in contrast to the sturgeon larval cell line (AOXlar7y from Atlantic sturgeon), which increased in size at higher passages [7]. Viability remained above 80–90%, indicating good adaptation and suitability for long-term storage in biobanks. The cell lines exhibited fibroblastic (RBC-ABAlar9 and 11), epithelial (lines 8 and 12), or mixed (line 7) morphologies, likely due to selection or spontaneous adaptations [21,22]. The cell lines were subsequently examined in detail using the Seahorse XF Cell Mito Stress Test and gene expression studies.

The gene expression largely resembled in vivo levels, remaining stable over 23 (RBC-ABAlar11) to 34 passages (RBC-ABAlar8). However, *gapdh*, *vim*, *col1a1*, and *pcna* demonstrated cell line-specific patterns. *Gapdh,* involved in glycolysis and stress responses, was elevated. In vitro systems are typically regarded as hyperoxic [23]. Yet the oxygen is difficult to estimate accurately and, depending on multiple factors, it may even fall to hypoxic levels. High glycolysis in cell lines, influenced by the Crabtree Effect or oxygen limitations, likely explains the higher *gapdh* expression in RBC-ABAlar cells, as an adaptation to in vitro cultivation [24]. Building on these metabolic differences, vimentin (*vim*), important for cell mechanics, motility, and wound healing [25], was increased in the AOXlar7y cells in response to thermal stress [9]. In RBC-ABAlar cells, *vim* was generally over-expressed compared to the tissue of origin. This suggests potential for in vitro adaptations arising from the mechanical properties of the culture vessel that differ from those in vivo. Extending these effects to extracellular matrix components, collagens are crucial fibrous components and are mainly produced in fibroblasts and osteoblasts [26,27]. *Col1α1* varied among the cell lines; RBC-ABAlar9 and 12 showed the highest expression, suggesting better reflection of in vivo *col1α1* expression. Morphological differences suggest variations in cell type composition between the fibroblast-like line 9 and the epithelial-like line 12, possibly reflecting different collagen-producing cells. Further analyses are required to clarify the cell-type distribution, inter-individual differences, and physiological fluctuations. Finally, with regard to proliferation, *pcna* fluctuated across passages and reached levels comparable to those in vivo, depending on the passage number and the cell line [9,28]. In the RBC-ABAlar cells, higher *pcna* expression is likewise associated with elevated metabolism and increased cell number.

Differences among the cell lines likely result from intrinsic cell heterogeneity, sibling level variation, and in vivo adaptation [29,30]. Furthermore, serum reduction from 20% FBS to 10% FBS after P10 may have contributed to some of the expression shifts observed [31]. Continuous characterization across passages remains essential to ensure the culture’s reliability.

### 4.2. Metabolic and MMP Analysis

The metabolic analysis of RBC-ABAlar cells indicated a predominantly aerobic metabolism, with comparable basal OCRs among the lines, similar to that of AOXlar7y [9].

Aquatic environments frequently experience hypoxia, requiring species-specific adaptations such as optimized oxygen uptake, metabolic suppression, and, in some fish, the use of alternative anaerobic end-products (e.g., ethanol) to avoid lactic acidosis under prolonged oxygen limitation [32,33]. The RBC-ABAlar cells were derived from larvae whose metabolism relies on yolk-sac-derived proteins, and amino acids are oxidized for energy. In this process, carbon skeletons enter the citric acid cycle for oxidative ATP production [34], resulting in low basal reliance on glycolysis, as previously shown for AOXlar7y cells [9]. However, RBC-ABAlar lines in this study exhibited different basal ECARs, with RBC-ABAlar8 having the highest and RBC-ABAlar9 the lowest, reflecting distinct metabolic dynamics. Such differences can be tissue and cell-type-dependent [35,36]. For example, Mito Stress Test with four different fish cell lines demonstrated bioenergetic differences, with SAF-1 (fibroblasts from *Sparus aurata*), and PHLC-1 (hepatocellular carcinoma from *Poeciliopsis lucida*) having higher metabolic rates than the two brain cell lines DLB-1 (derived from the brain of European sea bass) and FuB-1 (from the brain of Mummichog) [36].

The RBC-ABAlar cell lines responded differently to blockade of oxidative ATP synthesis by oligomycin [37], which is attributed to compensatory glycolysis, especially in RBC-ABAlar8. High ECAR mainly reflects glycolysis, but glutaminolysis can also indirectly influence it [38,39]. In senescent stem cells, impaired mitochondrial activity increases glutamine catabolism, whereas inhibition of glutaminolysis can improve respiration [40,41]. In teleost fish, glutaminolysis is an important pathway for ammonia production, and the gills likely play a key role in ammonia clearance during the yolk sac stage [42].

Interestingly, uncoupling of the electron transport from ATP synthesis with FCCP led to a further increase in ECAR in line 8, indicating stronger anaerobic glycolysis to meet energy demands, while oxidative respiration increased sharply in lines 7, 9, and 12. This pattern suggests a low reserve respiratory capacity of line 8, which is often associated with proliferative cells [36], whereas differentiated, post-mitotic cells typically have a high spare capacity. Hence, a low spare capacity does not necessarily indicate mitochondrial dysfunction, as high basal respiration or replication demands can deplete the reserve [43].

Rather, the MMP analysis revealed a high mitochondrial membrane potential in RBC-ABAlar8 cells, indicating healthy and functional mitochondria. The RBC-ABAlar8 metabolism appeared compatible with a proliferative profile that relies heavily on glycolysis and shows limited mitochondrial flexibility, rather than a FCCP-induced dysfunction. However, artifacts, especially cell-specific ones, cannot be entirely excluded: Oligomycin is known to inhibit FCCP-induced maximal respiration by 25% to 40% in highly glycolytic cells, depending on the cell type [44,45]. This inhibitory effect is reversed when the glycolytic metabolic pathway is minimized, either by using glutamine as the sole substrate for oxidative respiration or by inhibiting glycolysis [46]. Accordingly, the inhibitory effect of oligomycin on maximum respiratory capacity in RBC-ABAlar8 cells could be greater because these cells rely more heavily on ATP synthesis from anaerobic glycolysis than the other cell lines.

## 5. Conclusions

Fish cell lines are increasingly used as 3R-compatible alternatives to animal testing. Nevertheless, endangered species such as sturgeons remain underrepresented despite their urgent need for conservation. The established larval Siberian sturgeon cell lines from siblings showed common and distinct morphological, transcriptional, and metabolic features. They remained stable for at least 30 passages and are suitable for biobanking and interlaboratory transfer. These RBC-ABAlar lines enable analyses of individual and cell-type variability within a single genetic background, supporting more reliable assessments of stressor effects on larval health and development.

## Figures and Tables

**Table 1:** Gene-specific primers used in this study.

Gene Symbol	Official Name	NCBI Reference Sequence	Forward Primer (5′-3′)	Reverse Primer (5′-3′)	Product Size (bp)
Target Gene					
Regulation of glycolysis					
* gapdh *	Glyceraldehyde-3-phosphate dehydrogenase	XM_034042013.3	ACACCCGCTCAT-CAATCTTT	AGGTCCACGACTCTGTTGCT	80
Proliferation					
* pcna *	Proliferating cell nuclear antigen	XM_059013649.1	GCTGTGACGATCGAGATGAA	AACCAGAGCACACATGCTG	215
Pluripotency					
* pou5f1 *	POU domain, class 5, transcription factor 1	XM_058986684.1	GAGTCCCCT-CGTGATACAGG	CAGCACAGCCC-CTTTGATAC	150
Cytoskeleton					
* sptan1 *	Spectrin alpha chain, non-erythrocytic 1	XM_034927705.2	AGGGACACTTC-TCATCCGACAT	TGCAGCAGCCGCACACCCTT	106
* vim *	Vimentin	XM_034058632.3	GATTTCGCCTTGTCCGATGC	TTGGTGGTGCGT-TTTCCCTT	350
* col1a1 *	Collagen Type I Alpha 1 Chain	EU241879.1	CACCGAGGACGGTTACACAA	GTGCAATGTCG-ATGATGGGC	101
Stress-related genes					
* hsp70 *	Heat shock protein 70	XM_033996031.3	CCCGTGGAGAAGTCC	CCCGTTGAAGAAATCCTG	123
* hsf1 *	Heat Shock Transcription Factor 1	MH917287.1	CCAAGATTTGCTCGCACAGG	ACCAGCTGT-TTCCCAGTGTC	100
Reference genes					
* eef1a1 *	Eukaryotic Translation Elongation Factor 1 Alpha 1	XM_034004589.3	GGACTCCACTGAGCCACCT	GGGTTGTAGC-CGATCTTCTTG	90
* rpl6 *	Ribosomal Protein L6	HQ449564.1	GTGGTCAAACTC-CGCAAGA	GCCAGTAAG-GAGGATGAGGA	177

## Data Availability

All relevant data are provided within the manuscript.
